# Nationwide Drinking Water Sampling Campaign for Exposure Assessments in Denmark

**DOI:** 10.3390/ijerph15030467

**Published:** 2018-03-07

**Authors:** Denitza Dimitrova Voutchkova, Birgitte Hansen, Vibeke Ernstsen, Søren Munch Kristiansen

**Affiliations:** 1 Department of Geography, National University of Singapore, 1 Arts Link, Kent Ridge, Singapore 117570, Singapore; 2 Geological Survey of Denmark and Greenland (GEUS), 8000 Aarhus C, Denmark; bgh@geus.dk; 3 Geological Survey of Denmark and Greenland (GEUS), 1350 Copenhagen K, Denmark; ve@geus.dk; 4 Department of Geoscience, Aarhus University, 8000 Aarhus C, Denmark; smk@geo.au.dk

**Keywords:** sampling design, drinking water supply, groundwater, inorganic composition, iodine, public health, exposure assessment

## Abstract

Nationwide sampling campaign of treated drinking water of groundwater origin was designed and implemented in Denmark in 2013. The main purpose of the sampling was to obtain data on the spatial variation of iodine concentration and speciation in treated drinking water, which was supplied to the majority of the Danish population. This data was to be used in future exposure and epidemiologic studies. The water supply sector (83 companies, owning 144 waterworks throughout Denmark) was involved actively in the planning and implementation process, which reduced significantly the cost and duration of data collection. The dataset resulting from this collaboration covers not only iodine species (I^−^, IO_3_^−^, TI), but also major elements and parameters (pH, electrical conductivity, DOC, TC, TN, F^−^, Cl^−^, NO_3_^−^, SO_4_^2−^, Ca^2+^, Mg^2+^, K^+^, Na^+^) and a long list of trace elements (*n* = 66). The water samples represent 144 waterworks abstracting about 45% of the annual Danish groundwater abstraction for drinking water purposes, which supply about 2.5 million Danes (45% of all Danish residents). This technical note presents the design, implementation, and limitations of such a sampling design in detail in order (1) to facilitate the future use of this dataset, (2) to inform future replication studies, or (3) to provide an example for other researchers.

## 1. Introduction

The aim of this technical note is to provide a thorough account on the design of a nationwide drinking water sampling campaign implemented in Denmark in 2013. The main purpose of the sampling campaign was to inform about the spatial variation of iodine concentrations and speciation in Danish drinking water. Iodine is essential element that plays important role in the metabolism and early development of humans, but there was limited information on the local and regional variability of iodine species in Danish drinking water prior to our study [1]. Even though the main focus was on iodine, the resulting dataset can be used further for drinking water exposure assessments on other elements (e.g., lithium and strontium [2]) and following epidemiological studies (e.g., [3,4,5]). The methodology was briefly described in [1,2] with a focus on only few of the used analytes. In contrast, this technical note provides information on the entire dataset (major part of which has not been reported yet in peer-review publications). Here, we also elaborate on the design with specific focus on sampling site location choice and implementation limitations. The design details and the reasoning behind them may serve as a guide to others who are interested in conducting nationwide drinking water sampling for exposure studies or epidemiological analyses in Denmark and beyond. The existing drinking-water iodine studies rarely report selection criteria for drinking water sampling locations in satisfactory detail that limits the comparability between existing studies. The studies of Lv et al. [6] and Shen et al. [7] are exceptions with this respect, as there is enough detail for the water sampling to be reproducible and spatially representative. However, the highly cited studies on iodine in Danish drinking water preceding our sampling campaign lack this thoroughness: Pedersen et al. [8] took tap water samples from laboratories spread through Denmark (*n* = 55, locations only shown on low resolution map); Andersen, Petersen, and Laurberg [9] collected samples from Danish waterworks (*n* = 22), choosing them to verify low and high drinking water iodine contents when compared with their previous study [8]; Rasmussen, Larsen, and Ovesen [10] collected tap water from what they denoted as “*41 evenly distributed localities in the country*” which were neither listed nor mapped. Another major drawback of these iodine-drinking water sampling campaigns was that they did not account for the specifics of Danish drinking-water supply system, which we have addressed in our work. Therefore, in this technical note, we first provide some background on Danish drinking water supply to set the scene, and then we elaborate on the design and implementation of this drinking water sampling campaign. The dataset resulting from our drinking water campaign has so far been used in several studies [1,2,3,4,5] (status March 2017). We foresee that the details provided in this technical note will facilitate the future use of this “historical” dataset and will inform potential replication studies in Denmark.

## 2. Design

### 2.1. Drinking Water Supply in Denmark

Denmark is relatively small country (about 43,000 km^2^), with about 5.6 million inhabitants. Danish drinking water supply relies entirely on groundwater. The present Danish landscape is shaped mainly since the Weichselian glaciation and most of the Danish primary and secondary aquifers consist of Quaternary or Miocene sand and gravel or Paleocene to Late Cretaceous chalk and limestone. The spatial variability of groundwater composition with focus on iodine is discussed further in [11,12]. Danish households rely greatly on the water supply system, bottled water consumption in 2013 was 22.8 L/cap (0.127 million m^3^) [13], which was amongst the lowest in Europe and below the global average (30 L/cap [9]). Danish drinking water supply is characterized by decentralized structure of more than 2600 waterworks, with annual groundwater abstraction of about 400 million m^3^ per year [14], spread across the country. About 72% of the active waterworks have annual abstractions of <0.1 million m^3^, whereas about 3% are abstracting >1 million m^3^ [14]. Most of the Danish waterworks abstract groundwater from multiple wells or even multiple well sites. In some cases, groundwater from different aquifers is pumped up and mixed together before it gets to the treatment facilities. The raw groundwater undergoes simple physical and chemical treatment at the waterworks, consisting of aeration and sand filtration only. However, 74 waterworks (out of the >2600) with annual water production of about 50.47 million m^3^ have obtained permits to use some sort of advanced water treatment Table 1 [15]. Neither chlorination, nor ozone treatment are used, according to [15]. 

### 2.2. Sampling Site Selection

There were few initial limitations of the sampling design in relation to the scale and the nature of the study. Sampling all of the active waterworks was cost-inefficient, thus the number of sampling points was initially limited to about 180 (based on budget limitations and labor-time). As the focus of the study was on the spatial variability of iodine in drinking water, the campaign consisted of a single sampling event. The snap-shot nature of this sampling campaign was partially overcome by studying the short term temporal variability at one of the large waterworks supplying Copenhagen area (8 samples for two week period) (see Supplementary Data accompanying [1]). The results were inconclusive; however, previous investigations have pointed to relatively stable conditions for iodine and lithium [1,5]. Studying the temporal variability was not in the scope of our study, so this dataset limitation should be kept in mind if/when the dataset is used by others. The stability of regulated water quality parameters in raw groundwater and treated drinking water can be further evaluated based on the mandatory data reports to Jupiter database (Iodine is not part of the regular quality checks). Jupiter is the Danish public nationwide geological and hydrological database, which is maintained by the Geological Survey of Denmark and Greenland (GEUS) (www.geus.dk/DK/data-maps/jupiter/) and serves as an integrated information system for geology, groundwater, and drinking water [16]. Details on the access to data in the Jupiter database from different users and on data flow to and from the database can be found in [16].

**Figure 1:**
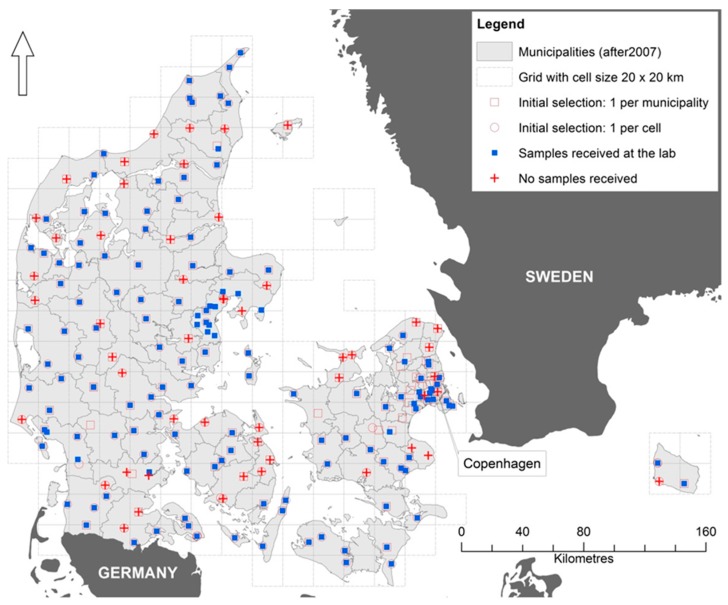
Sampling point location. Symbols: initial waterworks selection, based only on the two selection criteria (*n* = 181): 1st selection criteria (red empty square), 2nd criteria (red empty circle); waterworks which sent samples to the lab (*n* = 144) (blue square); contacted waterworks which did not participate i.e., no answer; negative answer; answered positively, but samples were not received at the lab (red cross) (note: the arrow indicates North direction).

The sampling site selection was based on data extracted from the publicly available Jupiter database (maintained by Geological Survey of Denmark and Greenland, GEUS) on 6 December 2012, consisting of the postal addresses, geographic coordinates, and annual abstraction volumes of all public waterworks (either privately or publicly owned) each supplying more than nine households. Based on this data, about 397 million m^3^ groundwater abstraction volumes were reported for 2010 by 2585 waterworks supplying more than nine households. Two selection criteria were adopted (Figure 1):

1. the largest waterworks in each municipality polygon (*n* = 99), and
2. the largest one in each grid cell (*n* = 189, 20 × 20 km) were selected.

The sampling campaign aimed to be representative for the drinking water (quality) supplied to as large proportion of the Danish population as possible. At the time of designing this campaign there was no readily available data on supplied area/population at the level of each waterworks, so the waterworks’ size, based on groundwater abstraction volumes for 2010, served as a proxy. The limited information on spatial variability of iodine in drinking water lead us to the second selection criteria, as to allow a geographically even coverage. When combining these two selection criteria, we identified 181 waterworks, from which only 27 had annual abstraction volumes <0.2 million m^3^. The 2010 groundwater abstraction volumes for none of the waterworks located in Sydjurs and Stevns municipalities were reported to Jupiter database, thus, 2007 data was used for these two municipalities instead. This added 5 more waterworks to the initial selection, resulting in a list with 186 waterworks representing about 40% of the annual groundwater abstraction for drinking water purposes in Denmark. This coverage was considered acceptable for the purposes of our study, so we proceeded with contacting the companies owning these waterworks, so we could obtain sampling permissions and further information. 

We incorporated feedback provided by some of the large water supply companies and adjusted the sampling site selection as to reflect the supply system complexity in some highly urbanized areas. Few companies advised us in inclusion of additional waterworks, as typically the supply of urbanized areas relies on more than one waterworks. Further corrections were needed also due to categorizing issues in the Jupiter data used for the initial selection: some of the ID numbers corresponded to single well sites (not the waterworks treatment facilities as usual), because some of the large waterworks report abstraction volumes for each well site separately to Jupiter. For these identified misclassification cases, the overlying waterworks (treatment facilities) were added to our selection list instead. After these adjustments, the final selection resulted in 189 sampling points all representing single waterworks, see Figure 1. 

### 2.3. Sampling

**Figure 2:**
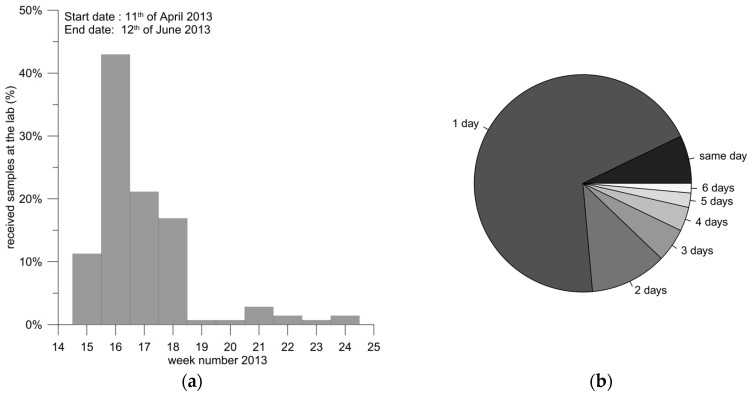
(**a**) Timing of samples received at the laboratory (%) indicating that the largest part of the sampling was conducted between weeks 15 and 18 (week 14 in 2013 started on 1 April); (**b**) Shipping periods: proportion of samples received at the lab on the same day of sampling or 1–6 days later.

**Figure A1:**
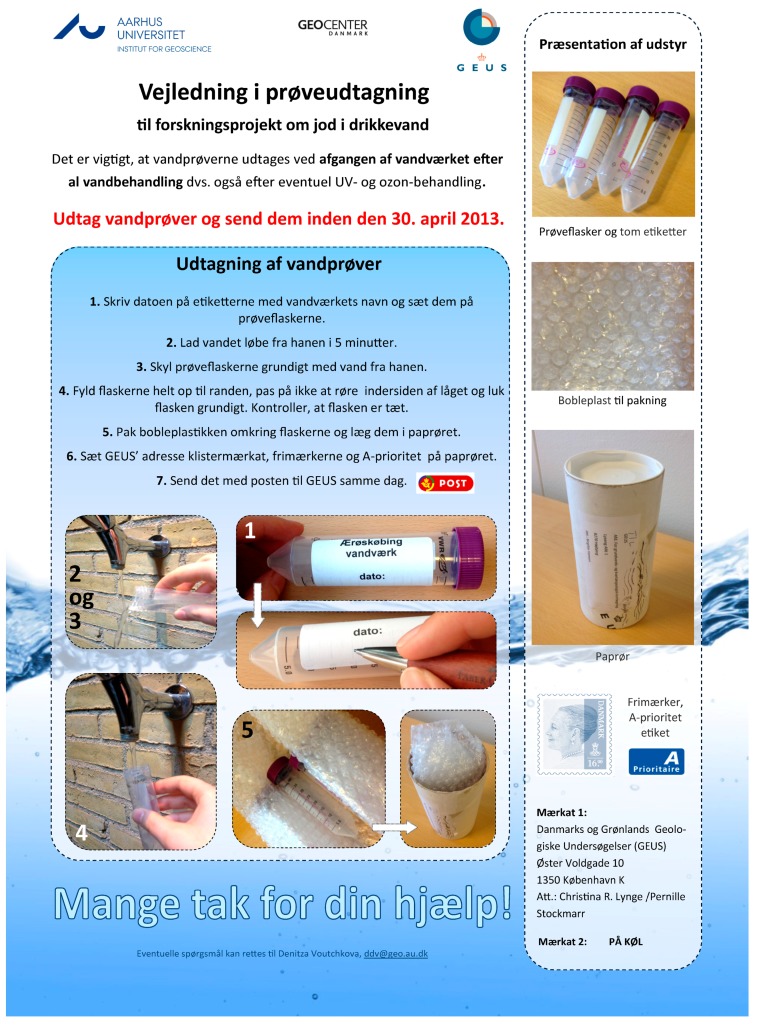
Manual for drinking water sampling sent to the participating waterworks (in Danish only); for translation see Box A1.

The sampling process was logistically challenging due to its spatial coverage. Since our study was aiming to obtain a snap-shot of the drinking water quality, prolonged sampling period was undesirable. After considering the practical challenges and estimating the time and resources needed, it was decided that a reasonable compromise is to involve the waterworks in the sampling process itself. Therefore, invitations for participations were sent to each identified owner of the selected waterworks. All who accepted our invitation received sampling materials, including the suit of sampling bottles and a manual describing the sampling procedure (Appendix A). We specified in the manual that the sampling point should be right after the treatment procedures and before the water leaves the facility to be supplied to consumers (at exit waterworks). Each sample consisted of four plastic bottles (i.e., centrifuge tubes, Figure A1) filled with treated drinking water. Field duplicates were planned randomly for 10% of the participating waterworks (duplicate sample consisted of eight bottles). Immediately after sampling the bottles were to be send to the Inorganic Lab at GEUS (Copenhagen, Denmark) by post. The samples were refrigerated at 4 °C degrees upon their arrival to the laboratory and were analyzed as soon as possible. To avoid overloading of the laboratory, the sampling packages were dispatched in two batches with two weeks difference in the beginning of April 2013. Despite this, most of the samples were received in the first four weeks of the period between 11 April and 12 June 2013 (Figure 2). A total of 76% of the samples were received at the lab the same day or a day after the sampling event (Figure 2).

### 2.4. Additional Data Collection

Questionnaires with mixture of closed and open questions were also sent to the contact persons at the participating waterworks in regards to the groundwater extraction well sites, treatment procedures, and drinking water supply and export (Appendix B). Materials in any form or links with relevant online sources of reliable and updated information about the abstraction and supply areas were requested as well.

## 3. Implementation

### 3.1. Water Sampling

Positive answers for participation in this study were received for 80% of the selected waterworks (*n* = 152), while four waterworks (2%) responded negatively. For the rest of the waterworks (*n* = 33, 17.5%), there was no answer after follow-up e-mails and phone calls. Samples from 144 (95% of the 152 which agreed to participate) were received and analyzed at the laboratory. Two of the 152 waterworks refused to receive the sampling packages and another sampling package was returned empty (open without the water samples). Taking into account the voluntary and non-profit character of the participation, the success-rate (95%) is exceptionally high. The initial agreement for participation by one of the waterworks was changed after receiving the questionnaire (see Table A1). The explanation was that completing the questionnaire is too laborious (this case is included in the four that refused). It is suspected that the success rate of this campaign is also affected by the unreliable (not up-to-date, or wrong) contact information for some of the waterworks. Nevertheless, it is believed that higher success rate would most likely be achieved only if such sampling was part of a state monitoring regulated by law. Including the parameter of interest, i.e., here iodine, in the routine water-quality monitoring of the waterworks would be the optimal way of collecting data on nationwide scale which describes both the spatial and the temporal variation of water composition.

The spatial distribution of the drinking water dataset resulting of this sampling design is covering more or less evenly the entire country (Figure 1). The dataset (*n* = 144) represents waterworks abstracting annually about 175 million m^3^, accounting for 45% of the total groundwater abstraction for drinking water purposes by all publicly and privately owned Danish waterworks (*n* > 2600). Only six of the waterworks can be considered small (annual abstraction < 0.1 million m^3^), whereas 46 (31.9%) have annual abstractions >1 million m^3^ [1]. Figure 2 of Supplementary data accompanying [1] provides more information on the abstraction volumes distribution from each of the 144 waterworks).

### 3.2. Additional Data Collection

The questionnaire was filled in at least partially for 94 waterworks (92 of which sent also water samples, 64% of total). Information about groundwater catchment areas of the production wells and well sites was provided in various formats. The varying quality of these data prevented their direct use for the purposes of the study; however, data on the geology at the waterworks’ wells from Jupiter database (GEUS) was summarized for the needs of our data analyses [1].

The information on supply areas was provided by all 94 waterworks; however, the quality and the type of information also varied: written free description of aerial parts, hand drawn sketches, links to online GIS, reports with high resolution figures, digital geocoded data sources (e.g., AutoCad, MapInfo, AcrMap files). Geocoded digital data, which is considered the most reliable, was provided for only 28 of the waterworks. Until 2014, the information on supply areas of the waterworks was not present in a single publicly-accessible database/repository, but instead it was part of the municipalities’ strategy plans (in Danish “vandforsyningsplan”). However, Schullehner and Hansen [17] compiled a map with the water supply areas of 2852 waterworks covering the entire country by collecting and digitalizing data from various sources (incl. the digital geocoded data collected in this study). This map enables the spatial connection between water quality supplied by the Danish waterworks with the resident history, health, and economic-social status on a personal level for the entire Danish population (see example in [2]). 

Based on this map [17] and two different datasets on residency, it was estimated that about 45.3% of all residents (by 2008) and 42.7% of all households (by 2012) are supplied with drinking water by the 144 waterworks included in this sampling campaign [2].

## 4. Data and Data Accessibility

**Figure 3:**
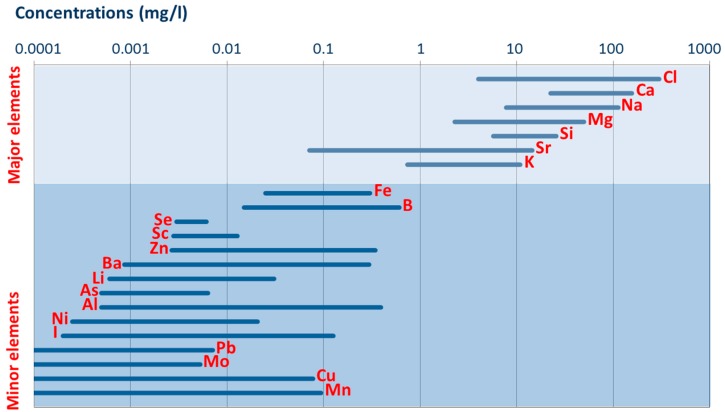
Concentration ranges for selected major and minor elements in drinking water from the 2013 sampling campaign of 144 major Danish waterworks revealing large concentration variability.

The data on drinking water chemistry from the individual water works are not reported here as this is beyond the scope of this technical note. However, we include an overview of concentrations’ level variation of major and minor elements (Figure 3), which may be of interest for future exposure, medical geology, or epidemiology studies. Methodological details for each of the analyzed parameters (including filtering, the analytical methods and detections limits) are provided in Appendix C.

Major elements and main parameters analyzed and included in the dataset (*n* = 13): pH, Electrical conductivity, Dissolved Organic Carbon, Total Carbon, Total Nitrogen, Fluoride, Chloride, Nitrate, Sulphate, Calcium, Magnesium, Potassium, and Sodium.

Minor elements that were analyzed and included in the dataset (*n* = 69, listed in alphabetic order): Aluminium, Antimony, Arsenic, Boron, Barium, Beryllium, Bismuth, Bromide, Cadmium, Cerium, Cobalt, Chromium, Caesium, Copper, Dysprosium, Erbium, Europium, Gallium, Gadolinium, Germanium, Gold, Hafnium, Holmium, Indium, Iron, Iridium, Iodide, Iodate, Iodine (total), Lanthanum, Lead, Lithium, Lutetium, Manganese, Mercury, Molybdenum, Niobium, Neodymium, Nickel, Osmium, Palladium, Phosphorus, Platinum, Praseodymium, Rhenium, Rhodium, Rubidium, Ruthenium, Samarium, Scandium, Selenium, Silicium, Silver, Strontium, Tantalum, Tellurium, Terbium, Thorium, Thallium, Thulium, Tin, Titanium, Tungsten, Uranium, Vanadium, Yttrium, Ytterbium, Zink, and Zircon.

The dataset obtained as part of this sampling campaign is partially available through the Danish open-access database Jupiter (GEUS). In general, all Jupiter data can be searched, mapped, visualized, and downloaded free of charge. The chemical analyses are associated with specific sample number, geographic location (via the waterworks identification number “JUPITER ID”), indication for the type of analyzed water (raw, treated, etc.), and specific sampling point. The data obtained as part of this study has been assigned unique attribute “JODGCP” (under project code). Due to the specifics of the individual agreements with some of the water supply companies involved in the sampling, however, the results from the 2013 sampling campaign will not be made readily available for download by “*unprivileged users*” (access classification, according to Figure 1A of [16]).

## 5. Conclusions

Drinking water quality monitoring is a mandatory part of EU countries’ legislation, as stipulated in the EU drinking water directive (Council Directive 98/83/EC of 3 November 1998 on the quality of water intended for human consumption). The Directive’s objective is to protect human health from the adverse effects of drinking water contamination. However, monitoring is only focusing on compounds with well-documented adverse effects, while the parameters with long-term adverse effects or naturally occurring elements with likely positive effects on human health are omitted. To study public health effects from such non-mandatory compounds, specific sampling campaigns are needed, and detailed account on their design and implementation is necessary to ensure the integrity and future reuse of the data. This technical note presents such an example of a successful involvement of the drinking water supply sector in a university research project focusing on iodine speciation in drinking water, but also including in total 82 elements and parameters for 144 waterworks in Denmark. The success rate and the spatial cover of the obtained drinking water chemistry data are surprisingly high taking into account the limitations of the methodology. The quality and quantity of the obtained data was considered appropriate with respect to the formulated goals of our studies [1,2]. However, for future use of this dataset, it is recommended to carefully consider its appropriateness with respect to the specific study goals. It should be kept in mind that this dataset represents a single sampling event and it is focused on spatial rather than temporal variability. Further, the chosen sampling methodology may have posed some problems with the reliability of some of the other hydrogeochemical data, especially with the volatile and redox sensitive elements. Naturally, treated Danish drinking water has low content of dissolved organic matter, so for the purposes of our study, sample preservation immediately after sampling was not needed. However, complexation may have affected samples with higher organic matter levels. 

## Figures and Tables

**Table 1:** Granted permits for advanced water treatment in Denmark (2007–2012). From those, number of active waterworks by 2012 and volumes of treated water produced yearly [15].

Type of Permit for Advanced Treatment	Permits (*n*) 2007–2012	Active Waterworks 2012	Treated Water (million m^3^/year)	Examples of Treatment Methods
Major components	43	29	13.26	addition of sodium hydroxide, precipitation by adding iron(II), flocculation with polyaluminium chloride
Inorganic trace elements	36	31	3.67	addition of iron chloride, iron sulphate or adsorption filters with iron oxide
Organic micro-pollutants	17	8	10.38	activated carbon filters, intense stripping
Microbiology	22	6	23.32	UV disinfection
*Total*	*118*	*74*	*50.47* ^1^	

^1^ the numbers don’t sum up precisely due to rounding.

## Appendix A

Box A1 Translation of the sampling manual from Danish (Figure A1). Guideline for sampling For the scientific project on iodine in drinking water. It is important that the water samples are taken at the exit of the waterworks, after all water treatment procedures, incl. UV or ozone treatment. Take samples and send them back before 30 April 2013. Sampling procedures Write the date on the label with the name of the waterworks and stick it to the sampling bottles. Let the water to flow for 5 min. Rinse the sampling bottle thoroughly with the water from the tap. Fill the sampling bottles until the top, be careful to not touch the inner side of the lid and close the sampling bottle tight. Check if the bottle is closed properly. Pack with the sampling bottles with bubble wrap and place them in the carton tube. Place the GEUS address sticker, the post stamps, the A-priority stamp on the tube. Send with the post the same day. Presentation of materials Sampling bottles and labels, bubble wrap, carton tube, post stamps, stickers with the address of the lab and “keep refrigerated”. Thank you for the help! For further questions, please contact Denitza Voutchkova, ddv@geo.au.dk.

## Appendix B

**Table A1:** Questionnaire (in Danish and translated in English).

**Danish Text (Original)**		**English Translation**
Indsamling af vandprøver til forskningsprojekt om jod i drikkevand Dette spørgeskema skal bruges i forskningsprojektet “Jod i det hydrologiske kredsløb i Danmark: betydning for menneskers sundhed” som foregår i et samarbejde mellem GEUS og Institut for Geoscience, Aarhus Universitet, og vi håber I kan besvare så mange af vore spørgsmål som muligt. Formålet med projektet er, at opnå ny videnskabelig forståelse af hvordan jod fordeler sig i dansk grund- og drikkevand samt belyse gavnlige effekter af jod i drikkevandet i forhold til folkesundheden. I alt 180 vandforsyninger fordelt over hele landet kontaktes og anmodes om at levere vandprøver til forskningsprojektet. For at opnå forståelse for jod og drikkevand, har vi brug for oplysninger om vandværket som vandprøven kommer fra. En del af de oplysninger vi beder om herunder er tilgængelige i GEUS’ Jupiter-database, men denne er desværre ikke opdateret med hvordan jeres vandværk fungerer når prøven udtages. Da resultaterne skal anvendes i et forskningsprojekt er det meget vigtigt, at vi har opdaterede og korrekte oplysninger. Så vi håber derfor, I har forståelse for, at vi beder om disse oplysninger som ellers synes offentligt tilgængelige. Udfyld venligst spørgeskemaet og send den til Denitza Voutchkova ddv@geo.au.dk senest XX Juni 2013.		National sampling campaign for the research project on iodine in drinking water This questionnaire will be used in the research project “Iodine in the hydrological cycle in Denmark: implications for human health”, which is collaboration between GEUS and the Department of Geoscience, Aarhus University, and we hope you could answer as many of the questions as possible. The purpose of the project is to obtain new scientific understanding of how iodine is distributed in Danish groundwater and drinking water, as well as to elucidate the beneficial effect of iodine in drinking water in relation to public health. A total of 180 waterworks across the country were contacted and asked to collect samples for this research project. In order to better understand the iodine variation in drinking water, we need additional information on the waterworks from which the samples come from. Some of the information we request below might be available in the Jupiter database, but might not be up-to date. As the results are going to be used in a research project, it is very important that we have the current and accurate information. So, we hope that you understand why we are asking for this information which is anyway publicly available. Please complete the questionnaire and send it to Denitza Voutchkova ddv@geo.au.dk the latest XX June 2013.
**N**	**Original Questions:**	**Translated Questions:**
**1**	Vandværkets navn:	Name of the waterworks:
**2**	Vandværkets adresse (UTM koordinater eller adresse inklusiv postnummer: X: Y: Address:	Address of the waterworks (UTM coordinates or address incl. post number): X: Y: Address:
**3**	Identifikations nummer (JUPITER/EAN):	Identification number (JUPITER/EAN)
**4**	Vandværkets behandlingsmetoder (ja/nej): (a) Kun iltning: (b) Iltning + filtrering: (c) UV eller ozonering: (d) andre metoder: hvis (b) specificer filtertypen: Åben/Lukket hvis (d) specificer metoder:	Treatment procedures in the waterworks (yes/no): (a) Only aeration: (b) Aeration + filtration: (c) UV or ozonation: (d) more sophisticated treatment: if (b) specify what type of filter: Open/Closed if (d) specify what type it is:
**5**	Har I for nylig ændret behandlingsmetoderne? (a) Ja (b) Nej hvis (a) Specificer hvornår: hvis (b) Hvilket år blev metoderne iværksat:	Have you changed the treatment recently? (a) Yes (b) No If (a) specify when: If (b) which year was implemented:
**6**	Har I ét eller flere rentvandstanke? (a) Ja (b) Nej hvis (a) Hvilket volumen har tanken/tankene (ca.):	Is/are there a pure water tank/s? (a) Yes (b) No If (a), what is the volume (ca.):
**7**	Fra hvor mange kildepladser indvinder I vand til dette vandværk?	How many well sites do you abstract water from?
**8**	Vil I venligst medsende information om indvindingsboringer og/eller-oplandsområder. (markere med et kryds hvilken/hvilke filtyper, der er tale om): (a) Indtegnet på kort: (b) Shape file: (c) AutoCAD file: (d) Vandforsyningsplan eller rapport: (e) Link til online information:	Could you please attach information about the abstraction wells and/or the groundwater catchment areas (mark with a cross what kind of file are you providing): (a) Drawn on a map (b) Shape file (c) AutoCAD file (d) Waterworks strategy plans or reports: (e) Link to online information
**9**	Hvor meget drikkevand produceredes (ca.): i 2010: i 2011:	How much drinking water was produced (ca.): In 2010: In 2011:
**10**	Hvor meget af det produceredes vand (%) forsyner (ca.): (a) Forbrugere: (b) Industri: (c) Andet: hvis (b) Specificer hvilken slags industri der især er tale om: hvis (c) Specificer det ”andet” som der især er tale om:	How much of the produced water (%) is supplied to the: (a) Consumers: (b) Industries: (c) Other: if (b) specify what kind of industry: if (c) specify what kind of other customer:
**11**	Vil I venligst sende os information om forsyningsområdet (hvilke byer eller områder) der normalt forsynes med vand fra vandværket. (markere med et kryds hvilken/hvilke filtyper, der er tale om): (a) Indtegnet på kort: (b) Shape file: (c) Autocad file: (d) Vandforsyningsplan eller rapport: (e) Link til online information:	Could you please attach information about which city/village or parts of the city are supplied by the waterworks normally. (mark with a cross what kind of file are you providing): (a) Drawn on a map (b) Shape file (c) Autocad file (d) Waterwork strategy plans or reports: (e) Link to online information
**12**	Importerer eller eksporterer I drikkevand? (a) Ja, vi importerer og eksporterer (b) Ja, vi importerer (c) Ja, vi eksporterer (d) Nej Hvis (a), (b), (c) specificer: hvorfra og hvor mange m^3^ (2010 og 2011): Hvortil og hvor mange m^3^ (2010 og 2011):	Do you import or export drinking water? (a) Yes, we import and export (b) Yes, we import (c) Yes, we export (d) No If (a), (b), (c), specify: from where and how much (2010 and 2011): to where and how much (2010 and 2011):
**13**	Dette spørgeskema er udfyldt af: Navn: Dato: E-mail: Telefonnummer:	This questionnaire was filled in by: Name: Date: e-mail: Phone number:
**14**	Ønsker I at modtage analyseresultaterne når alle analyser er færdige om ca. 1 år? (a) Yes (b) No	Would you like to receive the results when they are ready (in about 1 year)? (a) Yes (b) No
**15**	Andre kommentarer:	Other comments:
**Danish Text (Original)**		**English Translation**
Mange tak for hjælpen! **Hvis der er spørgsmål kontakt Ph.D. studerende Denitza Voutchkova ddv@geo.au.dk.** **Spørgsmål kan også rettes til lektor Søren M. Kristiansen, Institut for Geoscience på tlf. XXXX eller til seniorforsker Birgitte Hansen, GEUS, på tlf. XXXX.**		Thank you very much for the assistance! If you have any questions, please contact Ph.D. student Denitza Voutchkova ddv@geo.au.dk. Questions may also be addressed to Associate Professor Søren M. Kristiansen, Department of Geoscience, phone: XXX, or to Senior researcher Birgitte Hansen, GEUS, phone number: XXX.

## Appendix C

**Table A2:** Methodological details for all analyzed parameters and chemical elements.

Parameter or Element	Chemical Formula	Analytical Method	Filtering	Unit	Detection Limit
pH	pH	pH meter	no	pH	
Electrical conductivity	EC	Conductivity meter	no	µS/cm	
Iodide	I^−^	IC	Filtered 0.45 PES	I µg/L	0.9
Iodate	IO_3_^−^	IC	Filtered 0.45 PES	IO_3_ µg/L	1.4
Iodine	I	ICP-MS	Filtered 0.45 PES	I µg/L	0.2
Dissolved organic carbon	DOC	Total Organic Carbon Analyzer, Catalytic oxidation/NDIR method	Filtered 0.45 PES	C mg/L	0.5
Total inorganic carbon	TIC	Total Organic Carbon Analyzer, Catalytic oxidation/NDIR method	Filtered 0.45 PES	C mg/L	0.5
Total nitrogen	TN	Total Organic Carbon Analyzer/Chemiluminescence	Filtered 0.45 PES	N mg/L	0.25
Fluoride	F^-^	IC	Filtered 0.45 PES	mg/L	0.03
Chloride	Cl^−^	IC	Filtered 0.45 PES	mg/L	0.01
Bromide	Br^−^	IC	Filtered 0.45 PES	mg/L	0.02
Nitrate	NO_3_^−^	IC	Filtered 0.45 PES	mg/L	0.03
Sulfate	SO_4_^−−^	IC	Filtered 0.45 PES	mg/L	0.03
Calcium	Ca^++^	IC	Filtered 0.45 PES	mg/L	0.06
Potassium	K^+^	IC	Filtered 0.45 PES	mg/L	0.04
Sodium	Na^+^	IC	Filtered 0.45 PES	mg/L	0.03
Magnesium	Mg^++^	IC	Filtered 0.45 PES	mg/L	0.04
Silver	Ag	ICP-MS	Filtered 0.45 PES	mg/L	0.005
Aluminium	Al	ICP-MS	Filtered 0.45 PES	mg/L	0.1
Arsenic	As	ICP-MS	Filtered 0.45 PES	mg/L	0.01
Gold	Au	ICP-MS	Filtered 0.45 PES	mg/L	0.001
Boron	B	ICP-MS	Filtered 0.45 PES	mg/L	0.5
Barium	Ba	ICP-MS	Filtered 0.45 PES	mg/L	0.005
Berillium	Be	ICP-MS	Filtered 0.45 PES	mg/L	0.01
Bismuth	Bi	ICP-MS	Filtered 0.45 PES	mg/L	0.005
Calcium	Ca	ICP-MS	Filtered 0.45 PES	mg/L	0.5
Cadmium	Cd	ICP-MS	Filtered 0.45 PES	mg/L	0.001
Cerium	Ce	ICP-MS	Filtered 0.45 PES	mg/L	0.001
Chloride	Cl	ICP-MS	Filtered 0.45 PES	mg/L	50
Cobalt	Co	ICP-MS	Filtered 0.45 PES	mg/L	0.001
Chromium	Cr	ICP-MS	Filtered 0.45 PES	mg/L	0.05
Casium	Cs	ICP-MS	Filtered 0.45 PES	mg/L	0.001
Copper	Cu	ICP-MS	Filtered 0.45 PES	mg/L	0.001
Dysprosium	Dy	ICP-MS	Filtered 0.45 PES	mg/L	0.001
Erbium	Er	ICP-MS	Filtered 0.45 PES	mg/L	0.001
Europium	Eu	ICP-MS	Filtered 0.45 PES	mg/L	0.001
Iron	Fe	ICP-MS	Filtered 0.45 PES	mg/L	5
Gallium	Ga	ICP-MS	Filtered 0.45 PES	mg/L	0.001
Gadolinium	Gd	ICP-MS	Filtered 0.45 PES	mg/L	0.001
Germanium	Ge	ICP-MS	Filtered 0.45 PES	mg/L	0.001
Hafnium	Hf	ICP-MS	Filtered 0.45 PES	mg/L	0.001
Mercury	Hg	ICP-MS	Filtered 0.45 PES	mg/L	0.001
Holmium	Ho	ICP-MS	Filtered 0.45 PES	mg/L	0.001
Indium	In	ICP-MS	Filtered 0.45 PES	mg/L	0.001
Iridium	Ir	ICP-MS	Filtered 0.45 PES	mg/L	0.001
Potassium	K	ICP-MS	Filtered 0.45 PES	mg/L	2
Lanthanum	La	ICP-MS	Filtered 0.45 PES	mg/L	0.001
Lithium	Li	ICP-MS	Filtered 0.45 PES	mg/L	0.005
Lutetium	Lu	ICP-MS	Filtered 0.45 PES	mg/L	0.001
Magnesium	Mg	ICP-MS	Filtered 0.45 PES	mg/L	0.05
Manganese	Mn	ICP-MS	Filtered 0.45 PES	mg/L	0.005
Molybdenum	Mo	ICP-MS	Filtered 0.45 PES	mg/L	0.005
Sodium	Na	ICP-MS	Filtered 0.45 PES	mg/L	0.1
Niobium	Nb	ICP-MS	Filtered 0.45 PES	mg/L	0.001
Neodymium	Nd	ICP-MS	Filtered 0.45 PES	mg/L	0.001
Nickel	Ni	ICP-MS	Filtered 0.45 PES	mg/L	0.01
Osmium	Os	ICP-MS	Filtered 0.45 PES	mg/L	0.001
Phosphorus	P	ICP-MS	Filtered 0.45 PES	mg/L	2
Lead	Pb	ICP-MS	Filtered 0.45 PES	mg/L	0.005
Palladium	Pd	ICP-MS	Filtered 0.45 PES	mg/L	0.001
Praseodymium	Pr	ICP-MS	Filtered 0.45 PES	mg/L	0.001
Platinum	Pt	ICP-MS	Filtered 0.45 PES	mg/L	0.001
Rubidium	Rb	ICP-MS	Filtered 0.45 PES	mg/L	0.001
Rhenium	Re	ICP-MS	Filtered 0.45 PES	mg/L	0.001
Rhodium	Rh	ICP-MS	Filtered 0.45 PES	mg/L	0.001
Ruthenium	Ru	ICP-MS	Filtered 0.45 PES	mg/L	0.001
Antimony	Sb	ICP-MS	Filtered 0.45 PES	mg/L	0.001
Scandium	Sc	ICP-MS	Filtered 0.45 PES	mg/L	0.05
Selenium	Se	ICP-MS	Filtered 0.45 PES	mg/L	0.05
Silicium	Si	ICP-MS	Filtered 0.45 PES	mg/L	5
Samarium	Sm	ICP-MS	Filtered 0.45 PES	mg/L	0.001
Tin	Sn	ICP-MS	Filtered 0.45 PES	mg/L	0.001
Strontium	Sr	ICP-MS	Filtered 0.45 PES	mg/L	0.005
Tantalum	Ta	ICP-MS	Filtered 0.45 PES	mg/L	0.001
Terbium	Tb	ICP-MS	Filtered 0.45 PES	mg/L	0.001
Tellurium	Te	ICP-MS	Filtered 0.45 PES	mg/L	0.001
Thorium	Th	ICP-MS	Filtered 0.45 PES	mg/L	0.001
Titanium	Ti	ICP-MS	Filtered 0.45 PES	mg/L	0.005
Thallium	Tl	ICP-MS	Filtered 0.45 PES	mg/L	0.005
Thulium	Tm	ICP-MS	Filtered 0.45 PES	mg/L	0.001
Uranium	U	ICP-MS	Filtered 0.45 PES	mg/L	0.001
Vanadium	V	ICP-MS	Filtered 0.45 PES	mg/L	0.005
Tungsten	W	ICP-MS	Filtered 0.45 PES	mg/L	0.001
Yttrium	Y	ICP-MS	Filtered 0.45 PES	mg/L	0.001
Ytterbium	Yb	ICP-MS	Filtered 0.45 PES	mg/L	0.001
Zink	Zn	ICP-MS	Filtered 0.45 PES	mg/L	0.05
Zircon	Zr	ICP-MS	Filtered 0.45 PES	mg/L	0.001
